# CT-Guided Pulsed Radiofrequency at Different Voltages in the Treatment of Postherpetic Neuralgia

**DOI:** 10.3389/fnins.2020.579486

**Published:** 2020-12-18

**Authors:** Zhenkai Han, Tao Hong, Yuanyuan Ding, Shimeng Wang, Peng Yao

**Affiliations:** Department of Pain Management, Shengjing Hospital of China Medical University, Shenyang, China

**Keywords:** pulsed radiofrequency, postherpetic neuralgia, neuropathic pain, pain management, PRF parameters

## Abstract

**Background:**

Postherpetic neuralgia (PHN) is a form of long-lasting neuropathic pain that can severely affect patients’ quality of life. Pulsed radiofrequency (PRF) has been proven to be effective in treating PHN, but the optimal radiofrequency parameters are still not well defined. This retrospective study aimed to compare the efficacy and safety of CT-guided PRF at three different voltages for the treatment of PHN patients.

**Methods:**

This study included 109 patients with PHN involving the thoracic dermatome who were treated in the Department of Pain Management of Shengjing Hospital, China Medical University, from January 2017 to May 2019. They were divided into three groups based on the PRF voltage used: group A (45 V), group B (55 V), and group C (65 V). The PRF therapy (voltage 45, 55, and 65 V) was performed in all patients by targeting the thoracic dorsal root ganglion. After surgery, patients were followed at 3 days, 1 month, 3 months, 6 months, and 12 months. Observation at each follow-up included basic patient characteristics, visual analog scale (VAS), 36-Item Short Form Health Survey (SF-36) scores, patient satisfaction, complications, and side effects.

**Results:**

Visual analog scale scores decreased and SF-36 scores increased for all patients in the three groups at each post-operative time point (1, 3, 6, and 12 months; all *P* < 0.01). Pain relief, improvement in quality of life, and overall satisfaction were more significant for patients in group C than for those in groups A and B at the 3-, 6-, and 12-month follow-ups (all *P* < 0.05). Patients in group B had lower VAS scores and higher overall satisfaction levels than those in group A (both *P* < 0.01). A small number of patients from each group (*n* ≤ 3) experienced mild intraoperative and post-operative complications, which bore no relationship with group assignment (all *P* > 0.05). At post-operative day 3, patients in group C had skin numbness affecting a larger area than patients in the other two groups (both *P* < 0.05), but the differences were no longer statistically significant at day 30 after the operation. All patients experienced a drop in numbness area of more than 30% after surgery.

**Conclusion:**

Compared with PFR at 45 and 55 V, PFR at 65 V had superior efficacy in treating PNH, with a favorable safety profile.

## Introduction

Postherpetic neuralgia (PHN), a type of neuropathic pain, is the most common complication of shingles. Shingles occurs when the dormant varicella zoster virus activates, replicates, and propagates along an affected nerve, traveling down to the area of skin innervated by the nerve, and resulting in a painful blistering skin rash. At the same time, the affected nerves become inflamed and necrotized, followed by intense pain in the same area as the shingles rash (Yawn and Gilden, 2013; Koshy et al., 2018). If the pain persists for more than 1 month, the condition is called PHN. It is characterized by spontaneous pain, allodynia, and hyperalgesia. Long-lasting pain makes it difficult for patients to perform activities of daily living, work, and sleep. In severe cases, PHN patients will develop sleep disorders, anxiety, and even depression (Yang et al., 2019). As a result, their quality of life deteriorates dramatically.

Postherpetic neuralgia is intractable in that it changes the plasticity and sensory stability of pain neurons, causing peripheral and central sensitization of the pain sensation (Nalamachu and Morley-Forster, 2012). Treatment options for refractory PHN include oral medications (e.g., non-steroids, opiates, tricyclic antidepressants, and anticonvulsants) (Argoff, 2011), nerve root injection, paravertebral nerve block, continuous epidural analgesia, neurolysis, and implantation of stimulating electrodes (Lin et al., 2019). However, these treatment methods are not without disadvantages, including poor treatment outcomes, long duration, high recurrence rate, high risk of complications, and high costs (Guo et al., 2019).

Pulsed radiofrequency (PRF) is a novel therapeutic modality for pain relief and has been widely used in treating neuropathic pain. In recent years, it has been researched extensively, with controversial results on its efficacy (Vanneste et al., 2017). Many studies have shown that it is effective at relieving pain (Kim et al., 2017), although some studies reported limited efficacy and a high recurrence rate (Shi and Wu, 2016). We believe that the different inclusion criteria, small sample sizes, and inconsistent radiofrequency (RF) parameters used in these studies are responsible for the discrepancies between these research results.

Choosing optimal PRF parameters for PHN, in particular the voltage, remains controversial despite relevant research in this field (Luo et al., 2017). Here, we compared the efficacy and safety of PRF for PHN at three different voltages.

## Materials and Methods

### Patients

**FIGURE 1:**
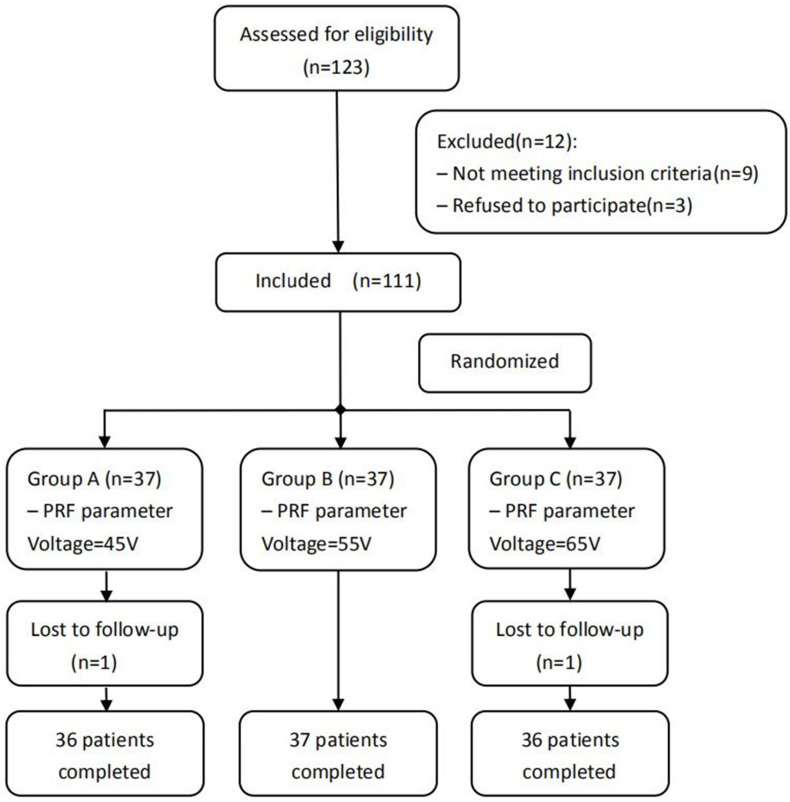
Schematic illustration of the study design. All 109 patients were included in the treatment.

A total of 109 patients with PHN affecting the thoracic dermatome treated in the Department of Pain Management of Shengjing Hospital, China Medical University, from January 2017 to May 2019 were included in our study. All patients included in the study are Chinese. They were all treated with PRF and were divided into three groups before treatment according to a computer-generated random number table: group A (45 V), group B (55 V), and group C (65 V) (Figure 1). This study was approved by the Ethics Committee of Shengjing Hospital, China Medical University (ID: 2017SP134K). All patients were informed of the risks and complications before surgery, and written informed consents were obtained.

### Inclusion Criteria

(1) Patients met the diagnostic criteria for PHN, i.e., skin pigmentation or scattered scars in the pain area, sensitivity to light touch, and frequent spontaneous intense burning, stabbing, or gnawing pain; (2) patients with PHN involving the thoracic dermatome; (3) pain that had lasted <6 months after the shingles rash had healed; (4) undesirable pain control with medications. the mean pain intensity score [visual analog scale (VAS)] ≥ 6 at 24 h before entering the group; and (5) age >40 years; (6) no nausea, vomiting, or dizziness before randomization.

### Exclusion Criteria

(1) Localized infection at the treatment site; (2) coagulation dysfunction; (3) severe cardiopulmonary insufficiency (American Society of Anesthesiologists, ASA physical status classification grade ≥3), or other major systemic diseases; (4) severe thoracic/lumbar spinal stenosis, compression fractures, or scoliosis; (5) mental illness; unwillingness to accept any possible operation-related complications; refusal of the operation itself; and (6) history of severe liver and kidney dysfunction or history of severe cardiopulmonary disease, pregnancy, and history of drug abuse.

### Surgical Methods

**FIGURE 2:**
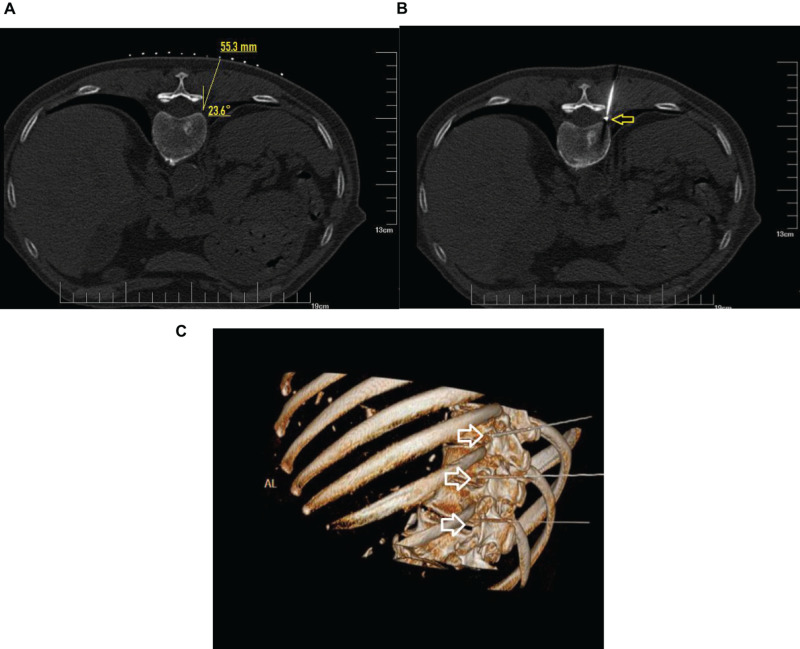
CT guidance: CT plain scan and 3D reconstruction. **(A)** Under CT guidance, a safe route was chosen to avoid injury to the vessel, pleura, and lung. The puncture point and puncture angle were clearly defined by computer. **(B)** CT scan showed that the radio frequency needle was located at the spinal nerve root on the left side and adjacent to the pleura and lung as shown by arrow. **(C)** The puncture needles of the three segments of the thoracic vertebrae were located at the intervertebral foramen as shown by the arrow.

The patient was placed in a prone position on the CT imaging bed with a soft pillow placed under the stomach area. ECG monitoring was performed continuously during the whole operation. Only the operation site was exposed, while other body parts were covered with lead safety clothing to afford radiation shielding protection. The negative electrode plate was attached to the flat surface of nearby muscles, and connected to the wires of the RF apparatus. Under CT guidance, the position of impaired nerve roots was located, and the route, angle, and depth of puncture were determined. After routine skin sterilization, draping, and local anesthesia with 0.5% lidocaine, the needle was inserted along the designated path. A CT scan was performed again to adjust the needle position so that the tip of the needle reached the dorsal root ganglion of the targeted nerve in the intervertebral foramen (Figure 2).

Then, electrical stimulation was performed to ensure coverage of the herpes area: (1) a sensation test, at 50 Hz, <0.2 V, to induce numbness in the innervated area of the nerve; (2) a neuromotor test, at 2 Hz, <0.5 V, to ensure there were no tremors over the innervated area. The position of the needle tip was adjusted until the stimulation test determined that the affected area was completely covered. After the dorsal root ganglion was attained, PRF treatment was performed according to the random number assigned to each patient. A staff member set up the parameters of the PRF apparatus for group A (45 V, 600 s), group B (55 V, 600 s), and group C (voltage 65 V, 600 s). When the operation was completed, the needle was withdrawn, and the puncture site was covered with dressings. Then, the patient was wheeled back to the ward. All procedures were performed by a doctor experienced in treating PHN with PRF.

### Follow-Up

To evaluate post-operative recovery, we conducted outpatient/telephone follow-ups or visited patients 1, 3, 6, and 12 months after the operation. Investigators participating in the follow-up activities were blind to the grouping or the PRF voltage used for each patient.

### Efficacy Evaluation

(1) VAS: Pain intensity was evaluated before and after treatment using VAS. Total scores ranged from 0 (no pain at all) to 10 (worst imaginable pain).
(2) 36-item Short Form Health Survey (SF-36): SF-36 was used to quantify health status in three domains, i.e., physical functioning, social functioning, and mental health. Each dimension was scored from 0 to 100, with higher scores indicating a better quality of life.

Five-point Likert scale: We used a five-pointTable 1 Likert scale to evaluate patient satisfaction.

**TABLE 1:** Five-point Likert scale.

Would you please evaluate the treatment effect after PRF surgery?	
5	Very satisfied
4	Satisfied
3	Same as before
2	Dissatisfied
1	Very dissatisfied

### Safety Evaluation

(1) Potential complications of the procedure: (1) Intraoperative complications included arrhythmia, nausea/vomiting, dizziness, being overstressed, or blood pressure drop. Puncture complications included pneumothorax and intraspinal or paravertebral hematoma. (2) Post-operative complications included localized redness and swelling, infection at the puncture site, and increased pain intensity. We recorded post-operative complications while the patients were still in the hospital and during follow-ups. For every complication, we offered prompt treatment and also recorded their progress in recovery.
(2) The area of skin numbness was marked by palpating the skin in conjunction with the patients’ own description. We then photographed the numbness area, and measured its size using the Image software. Relevant data were recorded before and after the operation.

### Statistical Methods

SPSS 19.0 statistical software was used to analyze the data. Measurement results that met the criteria of a normal distribution were expressed as the mean ± standard deviation ($\bar{x}$ ± sd). Data that did not conform to a normal distribution were expressed as the median ± interquartile range. Single factor analysis of variance was performed to compare the differences between groups when the data met the normal distribution and homogeneity of variance criteria. Otherwise, the Kruskal–Wallis test was performed. The χ^2^ test was used to compare enumerated data. Differences with *P* < 0.05 meant they were statistically significant.

## Results

### Pre-surgery Patient Characteristics

The patient characteristics recorded pre-surgery included sex, age, weight, disease duration, shingles distribution, pre-surgery pain intensity, and pre-surgery use of analgesics. No significant differences were found in these parameters between the three groups (all *P* > 0.05) (Table 2).

**TABLE 2:** Preoperative general characteristics of the patients (mean ± SD).

Parameters	Group A	Group B	Group C
Patients (*n*)	36	37	36
Gender (M/F, %)	17 (47.2)/19 (52.8)	17 (45.9)/20 (54.1)	16 (44.4)/20 (55.6)
Age (years, range)	67.67 ± 6.77 (54–83)	68.35 ± 9.47 (51–87)	68.19 ± 10.42 (49–83)
Weight (kg)	70.82 ± 7.36	71.29 ± 7.92	68.63 ± 8.72
Pain duration (month)	3.08 ± 1.07	3.13 ± 1.15	3.38 ± 0.93
Pain side (*n*, %)			
Left	18	20	17
Right	18	17	19
Preoperative VAS	7.54 ± 0.88	7.42 ± 0.64	7.67 ± 0.72
Preoperative drug dosage			
Gabapentin (g/day)	2.19 ± 0.63	2.29 ± 0.61	2.38 ± 0.84
Pregabalin (mg/day)	45.25 ± 7.69	42.75 ± 9.66	45.67 ± 12.08

### VAS Scores Before and After Surgery

**FIGURE 3:**
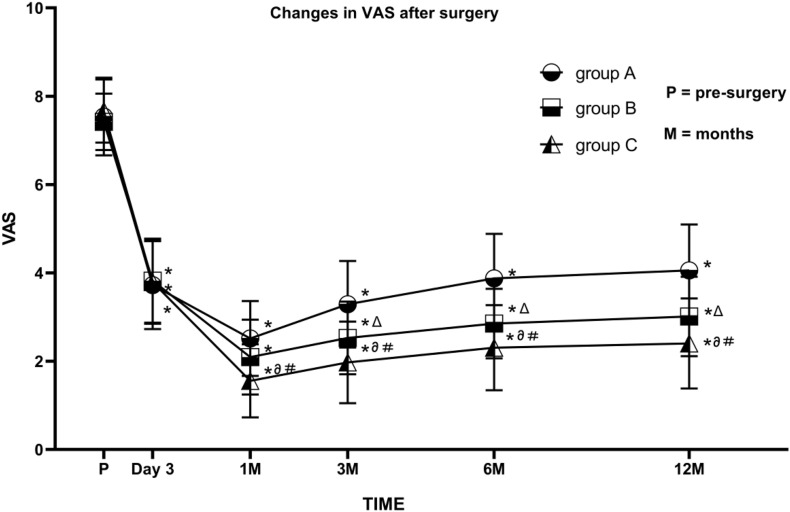
Comparison of visual analog scale (VAS) scores before and after PRF in the three groups. Group A (45 V PRF); Group B (55 V PRF); Group C (65 V PRF). Results are presented as mean ± SD. Compared to pre-surgery VAS, *P < 0.05; group B compared to group A, Δ*P* < 0.05; group C compared to group A, ∂*P* < 0.05; group C compared to group B, #*P* < 0.05.

With treatment, VAS scores decreased significantly in all three groups, reaching the lowest at 1 month after the operation. VAS scores were significantly lower than pre-surgery values for all patients from the three groups at each post-surgery observation time point (1, 3, 6, and 12 month; all *P* < 0.05). Patients in group C had lower VAS scores than those in the other two groups at the 3-, 6-, and 12-month follow-ups (all *P* < 0.05). For more than 80% of the patients, the VAS score was below 3 points. VAS values in group B were significantly lower than those in group A at some time points (*P* < 0.05) (Figure 3).

### SF-36 Scores Before and After Surgery

**FIGURE 4:**
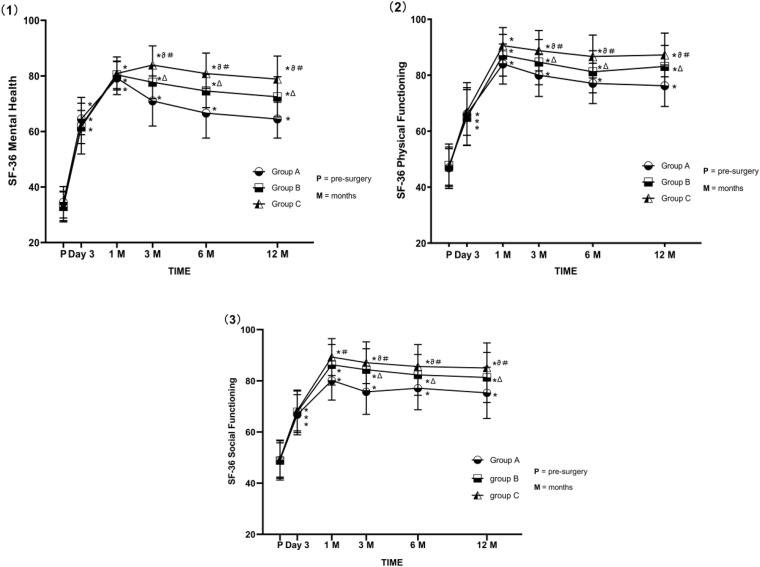
**(1–3)** Comparison of SF-36 (Physical Functioning, Mental Health, Social Functioning) scores before and after PRF in the three groups. Group A (45 V PRF); Group B (55 V PRF); Group C (65 V PRF). Results are presented as mean ± SD. Compared to pre-surgery SF-36 scores, *P < 0.05; group B compared to group A, Δ*P* < 0.05; group C compared to group A, ∂*P* < 0.05; group C compared to group B, #*P* < 0.05.

All patients had significantly increased SF-36 scores in domains including physical functioning, mental health, and social functioning at each post-surgery time point (all *P* < 0.05). Patients in group C had an average SF-36 score >80 at 1 month after surgery, significantly higher than those for group A and group B (both *P* < 0.05). Patients in group B had higher scores for physical functioning and mental health than those in group A during the long-term follow-ups (both *P* < 0.05); the exception was social functioning (Figure 4).

### Patient Satisfaction

We evaluated patients’ satisfaction with treatment outcomes with a five-point Likert scale. More than 80% of patients from each group were satisfied (four points) or very satisfied (five points) with the treatment. Patients in group C had higher satisfaction scores at the 3-, 6-, and 12-month follow-ups than those in group A (all *P* < 0.05). However, there were no significant differences in satisfaction scores between group B and group C at each post-operative time point (all *P* > 0.05) (Table 3).

**TABLE 3:** Likert scale five-point scoring system: global perceived effect; after PRF in the three groups.

	1M	3M	6M	12M
Group A	4.14 (4,1)	3.78 (4,2)	3.50 (4,1)	3.28 (3,1)
Group B	4.57 (5,1)Δ	4.35 (5,1)Δ	4.24 (4,1)Δ	4.11 (4,1)Δ
Group C	4.78 (5,0)**∂**	4.61 (5,1)**∂**	4.44 (5,1)**∂**	4.42 (5,1)**∂**

*Data expressed as the mean (median ± quartile range); group B compared to group A, ΔP < 0.05; group C compared to group A, ∂P < 0.05; group C compared to group B, #P < 0.05.*

### Safety Evaluation

During PRF treatment, a very small number of patients in each group (*n* ≤ 3) experienced transient arrhythmia, dizziness, nausea/vomiting, headache, or hypotension. With prompt intraoperative symptomatic treatments, these symptoms were relieved within 30 min. No pneumothorax or intraspinal/paravertebral hematoma occurred.

After PRF treatment, 2 of the 109 patients developed localized redness and swelling, which disappeared after the application of cold compresses for 2 days. No other complications occurred (Table 4). The above complications were not related to grouping (all *P* > 0.05).

**TABLE 4:** Adverse events about various voltage parameters PRF.

	Group A, 45 V *n* = 36, *n*	Group B, 55 V *n* = 37, *n*	Group C, 65 V *n* = 36, *n*
During the PRF therapy			
Arrhythmia	0	1	0
Dizziness	1*	1	0
Nausea and vomiting	1*	0	2
Headache	0	0	0
Hypotension	0	1	1
After the PRF therapy			
Local swelling	1	0	1
Infection	0	0	0
Worsening pain	0	0	0

*^∗^Same patient.*

### Numbness Area Before and After the Operation

**FIGURE 5:**
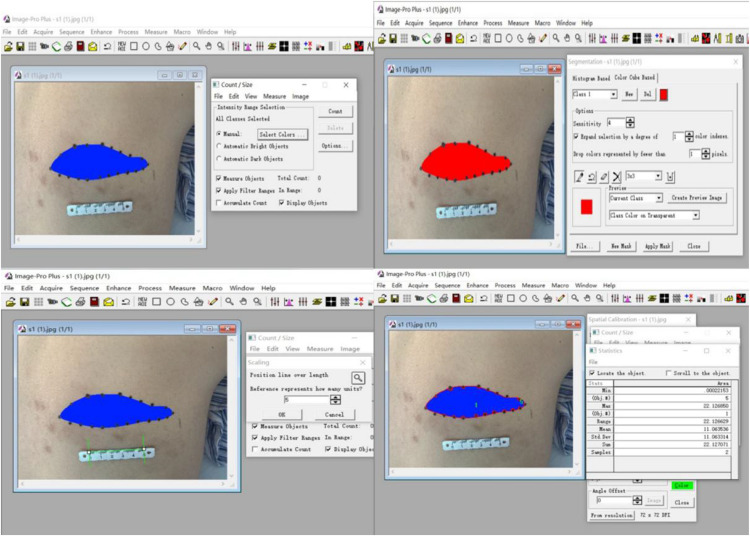
Measurement of the numbness area using Image-Pro Plus 6.0 software.

Calculation of the size of the numbness area innervated by the affected nerve is shown in Figure 5. There were no statistically significant differences in the sizes of the numbness areas between each group before the operation (all *P* > 0.05). Patients in group C had a larger area of numbness than those in the other two groups at 3 days after the operation (both *P* < 0.01), but these differences were no longer statistically significant during the 30-day follow-ups (all *P* > 0.05). All patients experienced a decrease of at least 30% in the size of the numbness area (all *P* < 0.01, Table 5).

**TABLE 5:** Numbness area in different voltages PRF.

	Pre-surgery (area, cm^2^)	3 days (area, cm^2^)	30 days (area, cm^2^)
Group A	44.15 ± 12.13	36.36 ± 13.32*	28.56 ± 14.27*
Group B	45.04 ± 11.62	41.05 ± 13.71	31.20 ± 12.12*
Group C	46.09 ± 11.26	53.80 ± 13.21*^∂#^	31.54 ± 11.86*

*Compared to pre-surgery numbness area, ^∗^P < 0.05; group B compared to group A, ΔP < 0.05; group C compared to group A, ∂P < 0.05; group C compared to group B, #P < 0.05.*

## Discussion

Postherpetic neuralgia is a very common neuropathic pain. Early and appropriate intervention can effectively relieve the pain and improve patients’ quality of life (Saguil et al., 2017). In our study, we offered PRF to all patients with PHN involving the thoracic dermatome at three levels of voltage. With treatment, all patients had significantly deceased VAS scores and increased SF-36 scores during the post-operative 12-month follow-up period, proving the desirable efficacy of PRF treatment. Despite the recurrence of pain in some patients from 3 to 12 months after the operation, most patients were satisfied with the treatment outcomes, which is consistent with previous research results (Kim et al., 2008; Makharita et al., 2018).

The mechanism by which PRF relieves PHN pain is not fully understood, The analgesic mechanism of PRF is unclear, but it is different from conventional radiofrequency. The radiofrequency current of PRF is not incessant. This energy transfer does not destroy the anatomical basis of pain impulse transmission and does not cause nerve damage and protein coagulation. It is currently believed that PRF is neuromodulation for pain relief (Ding et al., 2019; Vuka et al., 2020; Wu et al., 2020). The RF electrode produces a high-voltage electric field at the target nerve, and controlled heat is generated from ion friction, which is dissipated during the relatively long pause between pulses (Cahana et al., 2010). The “silent” phase of PRF allows sufficient time for heat elimination, generally keeping the target tissue below 42°C to avoid causing neuronal injury (van Boxem et al., 2008). PRF creates an electric field effect that modulates the over-active synaptic transmission associated with chronic pain and changes the structure of nerve fibers (Tanaka et al., 2010).

Models of neuropathic pain in the rat showed that the activity of microglia in the spinal dorsal horn was decreased and the pERK expression was downregulated, which affects the activity of ion channels of the peripheral nerve cells such as the P2X ligand-gated ion channel 3 and inhibits the peripheral sensitization of PHN (Cho et al., 2013; Liu et al., 2015; Fu et al., 2019). Research on central sensitization has found that PRF increases the expression of activating transcription factor 3 in pain conduction C and Aδ fibers, which activates the descending pain inhibitory system in the brainstem to achieve analgesic effects (Huang et al., 2017). PRF can also increase the levels of brain-derived neurotrophic factor and glial cell-derived neurotrophic factor, decrease the level of calcitonin gene-related peptide, and increase the expression of β-endorphin precursors (Hailong et al., 2018; Ren et al., 2018). Recent research results reveal that PRF induces a mild electroporation effect, causing a calcium uptake in the mechanism of chronic pain treatment (Mercadal et al., 2020).

SF-36 is a very popular instrument for evaluating health-related quality of life, and it can be used to evaluate PHN patients’ physical and mental health after PRF treatment. It has three domains including physical functioning, social functioning, and mental health (Lam et al., 2005). In our study, we found that patients who received PRF at a higher voltage had higher SF-36 scores, especially in the subscales of physical functioning and mental health. In addition, we used the easily administered five-point Likert scale to evaluate patient satisfaction (Harland et al., 2015). We found that most patients were satisfied with the treatment outcomes, and patients in group C had greater satisfaction.

A higher output voltage and the resulting electric field intensity can achieve better efficacy. This is because high RF voltage increases the output energy of the electric field effect in the target nerve. PRF efficacy may be positively correlated with the amount of energy received by local tissues (Teixeira and Sluijter, 2006). However, the upper limit of RF voltage that can be applied is determined by the patient’s tolerance and the incidence of complications. Consistent with previous reports, we found, through long-term follow-ups, that patients in group C had significantly lower VAS scores, better quality of life, and higher satisfaction scores than those from the other two groups (Luo et al., 2013).

In this study, less than 10% of patients had transient arrhythmia, dizziness, nausea/vomiting, or hypotension during PRF treatment, and all showed immediate relief after symptomatic treatments. We believe these adverse reactions occurred because the patients were nervous, and there was no correlation between these complications and PRF output voltage. After PRF treatment, two patients experienced localized hematoma for a short time, which was related to the puncture procedure. No serious complications such as pneumothorax and intraspinal or paravertebral hematoma occurred during the whole operation. In treating thoracic PHN, many researches considered the precision of CT guiding, which can effectively and safely avoid damaging the pleura and lung tissues. Patients who received high-voltage PRF treatment initially had a larger numbness area, which was caused by the increased local temperature induced by the higher PRF output voltage. However, the numbness area gradually became smaller during follow-ups and was significantly smaller than the pre-surgery value.

In this study, we found that the tissue temperature near the electrode rose beyond the critical level of 42°C and could be as high as 50°C when the PRF voltage was increased to 65 V. In addition, we only included patients with PHN involving the thoracic dermatome in our study, which avoided outcomes such as motor nerve injury and organ dysfunction. Consistent with findings by Heavner et al. (2006), we found that protein denaturation during PRF only occurred when the temperature reached 60°C. The short-term increase in the size of the numbness area may be related to enhanced blocking of Aδ and C fiber conduction due to the increased local electric field energy produced by the high RF output voltage. Therefore, it is safe to perform PRF at 65 V with no risks of long-term complications.

However, our study was not without limitations. First, the sample size in our study was small, and multi-center large-sample studies are needed to further verify our findings. Second, we did not perform subgroup analysis based on patients’ disease stage and age. We continue to collect cases and intend to refine the groupings and get more in-depth results in the future. Lastly, we did not increase the PRF voltage beyond 65 V. If the voltage was further increased, the temperature could not be maintained below 50°C, which might cause protein degeneration and nerve damage; meanwhile, the purpose of our study is also to distinguish It from the CRF technology. In future studies, we will further refine the PRF voltage parameters and expand parameter range as long as it can be tolerated by the patients.

In conclusion, we found that PNH patients who received PRF had significantly alleviated pain, improved quality of life, and high overall satisfaction. PRF at a higher voltage significantly improved treatment outcomes without increasing the risk of intraoperative and post-operative complications. Therefore, it is safe and effective to treat patients who have PNH involving the thoracic dermatome with PRF at 65 V.

## Data Availability Statement

The original contributions presented in the study are included in the article/supplementary materials, further inquiries can be directed to the corresponding authors.

## Ethics Statement

The studies involving human participants were reviewed and approved by Ethics Committee of Shengjing Hospital of China Medical University. The patients/participants provided their written informed consent to participate in this study. Written informed consent was obtained from the individual(s) for the publication of any potentially identifiable images or data included in this article.

## Author Contributions

ZH, PY, and YD: conception and design of the study. ZH, TH, and SW: acquisition of data. ZH and TH: data analysis. ZH and PY: drafting the manuscript. All authors contributed to the article and approved the submitted version.

## Conflict of Interest

The authors declare that the research was conducted in the absence of any commercial or financial relationships that could be construed as a potential conflict of interest.
